# Enhancement of Bone-Forming Ability on Beta-Tricalcium Phosphate by Modulating Cellular Senescence Mechanisms Using Senolytics

**DOI:** 10.3390/ijms222212415

**Published:** 2021-11-17

**Authors:** Xinchen Wang, Yoshitomo Honda, Jianxin Zhao, Hidetoshi Morikuni, Aki Nishiura, Yoshiya Hashimoto, Naoyuki Matsumoto

**Affiliations:** 1 Department of Orthodontics, Osaka Dental University, 8-1 Kuzuhahanazonocho, Hirakata 573-1121, Osaka, Japan; cindyoops1126@hotmail.com (X.W.); jianxinzhao@hotmail.com (J.Z.); morikuni@cc.osaka-dent.ac.jp (H.M.); nishiura@cc.osaka-dent.ac.jp (A.N.); naoyuki@cc.osaka-dent.ac.jp (N.M.); 2 Department of Oral Anatomy, Osaka Dental University, 8-1 Kuzuhahanazonocho, Hirakata 573-1121, Osaka, Japan; 3 Department of Biomaterials, Osaka Dental University, 8-1 Kuzuhahanazonocho, Hirakata 573-1121, Osaka, Japan; yoshiya@cc.osaka-dent.ac.jp

**Keywords:** cellular senescence, beta-tricalcium phosphate, bone formation, senolytics

## Abstract

Various stresses latently induce cellular senescence that occasionally deteriorates the functioning of surrounding tissues. Nevertheless, little is known about the appearance and function of senescent cells, caused by the implantation of beta-tricalcium phosphate (β-TCP)—used widely in dentistry and orthopedics for treating bone diseases. In this study, two varying sizes of β-TCP granules (<300 μm and 300–500 μm) were implanted, and using histological and immunofluorescent staining, appearances of senescent-like cells in critical-sized bone defects in the calvaria of Sprague Dawley rats were evaluated. Parallelly, bone formation in defects was investigated with or without the oral administration of senolytics (a cocktail of dasatinib and quercetin). A week after the implantation, the number of senescence-associated beta-galactosidase, p21-, p19-, and tartrate-resistant acid phosphatase-positive cells increased and then decreased upon administrating senolytics. This administration of senolytics also attenuated 4-hydroxy-2-nonenal staining, representing reactive oxygen species. Combining senolytic administration with β-TCP implantation significantly enhanced the bone formation in defects as revealed by micro-computed tomography analysis and hematoxylin-eosin staining. This study demonstrates that β-TCP granules latently induce senescent-like cells, and senolytic administration may improve the bone-forming ability of β-TCP by inhibiting senescence-associated mechanisms.

## 1. Introduction

Bone defects attributed to periodontitis, trauma, surgery, or congenital malformations are a crucial clinical issue [1]. Currently, autogenous bone grafting is the gold standard clinical procedure for repairing bone defects, but the limited availability of donor bone hamper further operation [2]. Thus, various bone grafting materials are extensively investigated [3]. Calcium phosphate (CaP)-based scaffolds, such as beta-tricalcium phosphate (β-TCP) [4], alpha-tricalcium phosphate [5], hydroxyapatite [6], and octacalcium phosphate [7], have been studied as bone graft substitutes because of their high biocompatibility, biosafety, and long shelf life [8]. The β-TCP scaffold has occasionally been used in clinical practice for periodontal defects, as it exhibits excellent osteoconductive properties [9]. However, when compared with that of the autogenous bone graft, the osteogenic capacity of β-TCP is insufficient, thus limiting its further use [10]. Some studies have reported that β-TCP occasionally induces inflammation [11,12] and promotes the production of reactive oxygen species [13,14], thus contributing to the attenuation of osteoconductivity. Further elucidation on the bone-forming mechanism of β-TCP may widen its usage aspects.

Senescent cells undergo an irreversible cell-cycle arrest mechanism, are highly metabolically active, and secrete large amounts of various substances into the environment [15,16]. A variety of stresses can induce cellular senescence, including reactive oxygen stress, mechanical stress, glycation stress, DNA damage, etc. [17]. The pathways induced by these stress stimuli are transferred to a series of senescence inducing genes—mainly *Cdkn2a-p19*, *Cdkn1a-p21*, *Cdkn2b-p15*, and *Cdkn2a-p16,* eventually leading to the arrest of cell proliferation [18,19]. Stress-induced premature senescent (SIPS) cells exhibit the same features as cells undergoing replicative senescence, including morphological changes, activation of cell cycle arrest mechanisms, irreversible cell cycle arrest, DNA damage response, as well as secretion of a senescence-associated secretory phenotype [20]. The accumulation of senescent cells leads to the aging of tissues, which further results in tissue dysfunction [21,22]. Lately, a study on long bone growth in mice reported that cellular senescence mediates the toxic effects of prenatal dexamethasone exposure (PDE) [23]. It was found that inhibiting cellular senescence could treat PDE-induced bone growth retardation and promote bone formation [23]. Furthermore, a model of senescence induction has been created by using lipopolysaccharide-conjugated gelatins in rats [24]. It revealed that SIPS cells inhibit bone regeneration, and local administration of epigallocatechin gallate or oral administration of senolytics (a cocktail of dasatinib (D) and quercetin (Q), hereafter DQ) decreased this inhibition [25].

Senolytics are drugs that specifically target senescent cells rather than targeting apoptosis in non-senescent cells [26,27]. D and Q alone, or a combination of the two, are the most-studied senolytic drugs [28]—where D is an antineoplastic agent used in the treatment of acute lymphocytic leukemia, chronic granulocytic leukemia, and leukemic leukemia [29]; Q is a flavonoid with antioxidant, anti-inflammatory, immunoprotective properties, or anticancer activity [30]; and oral administration of DQ has been reported to discriminatively decrease senescent cells, moderate inflammatory cytokine secretion, relieve physiological dysfunction, and improve the functioning of various tissues associated with cellular senescence [31,32], including bone [33], fat [34], lung [35], the cardiovascular system [36], etc. However, it is still unclear whether the implantation of β-TCP induces senescent cells and how the removal of senescent cells affects bone regeneration of β-TCP. 

This study aimed to elucidate the appearance of senescent cells after implanting two different sizes of β-TCP in critical-sized bone defects of rat calvaria. We further investigated whether the oral administration of DQ affects the bone-forming ability of β-TCP. 

## 2. Results

### 2.1. Characteristics of β-TCP

**Figure 1:**
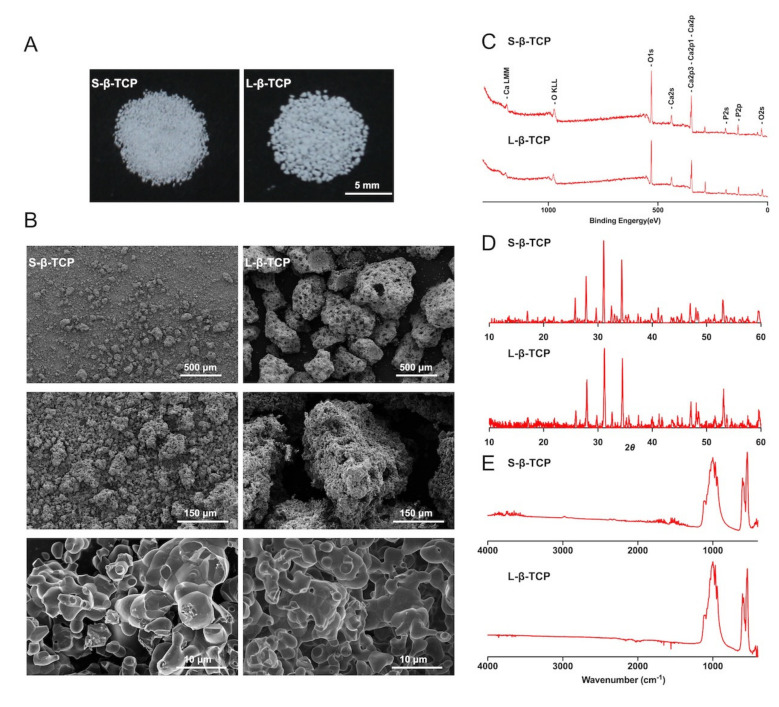
Beta-tricalcium phosphate (β-TCP) detections and measurements. (**A**) Macroscopic images of β-TCP granules; (**B**) field emission-scanning electron microscopic (FE-SEM) images of β-TCP granules with low and high magnification; (**C**) X-ray photoelectron spectroscopy (XPS) analysis of the β-TCPs; (**D**) X-ray diffraction (XRD) patterns of β-TCPs; (**E**) attenuated total reflection-Fourier transform infrared spectroscopy (ATR-FTIR) spectra recordings of β-TCPs.

To prepare the two different sizes of β-TCP, intact β-TCP granules were grained and sieved to smaller granules below 300 μm (hereafter designated as S-β-TCP) and larger granules from 300 μm to 500 μm (hereafter designated as L-β-TCP) (Figure 1A). Images captured utilizing a field emission-scanning electron microscope (FE-SEM) proved that these granules differed in size but had similar smooth surfaces (Figure 1B). Both S- and L-β-TCPs showed similar and typical spectra of β-TCPs on X-ray photoelectron spectroscopy (XPS), X-ray diffraction (XRD), and attenuated total reflection-Fourier transform infrared spectroscopy (ATR-FTIR) (Figure 1C–E) [37,38]. These spectra confirmed that the crystalline phase of β-TCP was unchanged even after the preparation.

### 2.2. SIPS Cells in Bone Defects after β-TCP Implantation

**Figure 2:**
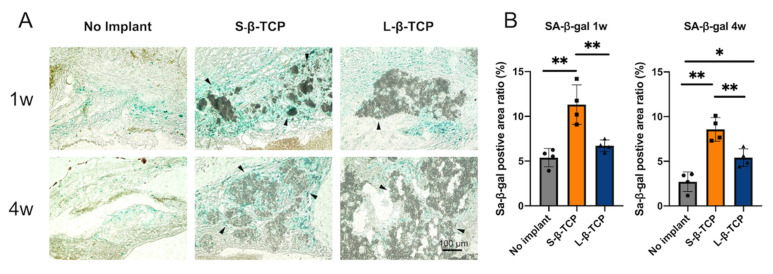
Identification of cellular senescence in bone defects at one week and four weeks after the operation. (**A**) Senescence-associated beta-galactosidase (SA-β-gal) staining images of bone defects (scale bar = 100 μm). Black triangles represent β-TCP granules; (**B**) SA-β-gal positive area ratio in the bone defect. Mean with standard deviation (SD) (*n* = 4). * *p* < 0.05, ** *p* < 0.01: One-way analysis of variance (ANOVA) with Tukey–Kramer method as post hoc test.

**Figure 3:**
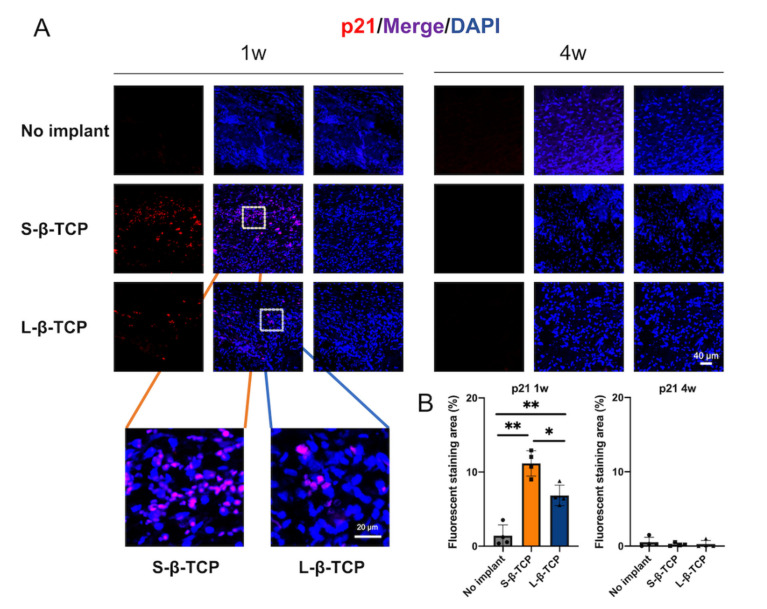
Identification of cellular senescence in bone defects at one week and four weeks after the operation. (**A**) Immunofluorescent images of bone defects stained with p21 antibody and 4′,6-diamidino-2-phenylindole (DAPI) (scale bar = 40 μm); (**B**) p21 quantified protein level at region of interest (ROI) in bone defect. Data are presented as the ratio of protein immunofluorescent area to nucleus area. Mean with SD (*n* = 4). * *p* < 0.05, ** *p* < 0.01: One-way ANOVA analysis with Tukey–Kramer method as post hoc test.

**Figure 4:**
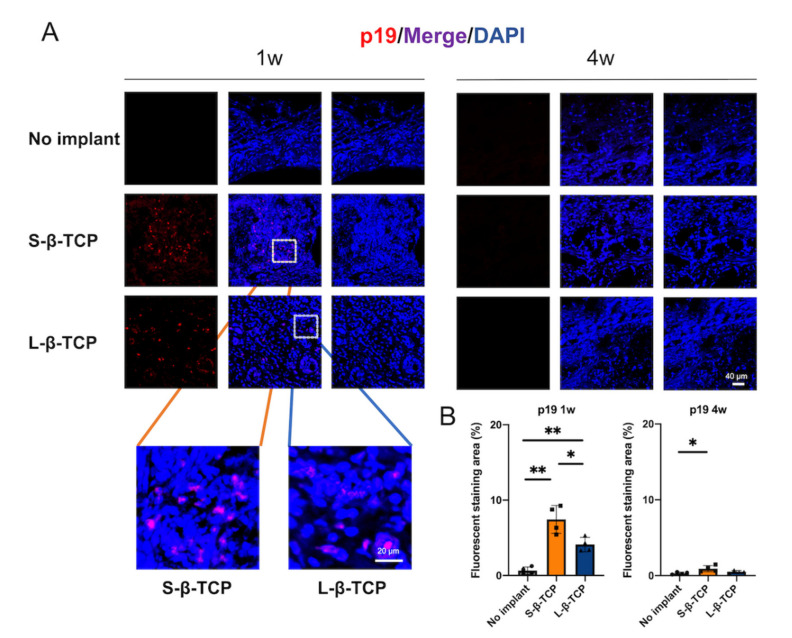
Identification of cellular senescence in bone defects at one week and four weeks after the operation. (**A**) Immunofluorescent images of bone defects stained with p19 antibody and DAPI (scale bar = 40 μm); (**B**) p19 quantified protein level at ROI in the bone defect. Data are presented as the ratio of protein immunofluorescent area to nucleus area. Mean with SD (*n* = 4). * *p* < 0.05, ** *p* < 0.01: One-way ANOVA analysis with Tukey–Kramer method as post hoc test.

**Figure 5:**
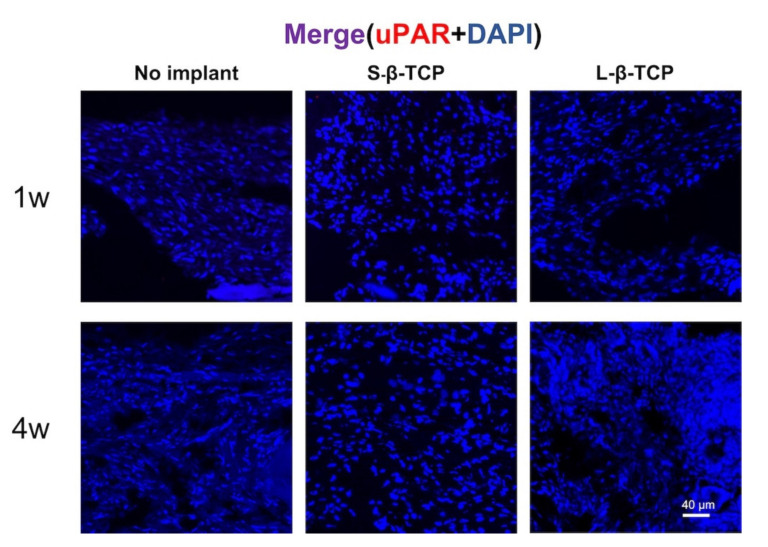
Identification of cellular senescence in bone defects. Immunofluorescent staining images of bone defects at one week and four weeks after operation stained with urokinase plasminogen activator receptor (uPAR) antibody and DAPI (scale bar = 40 μm).

Senescence-associated beta-galactosidase (SA-β-gal) is a conventional indicator for cellular senescence [39,40]. Recently, p21 and p19 have been recognized as markers to indicate cells in early senescence [41,42]. Bone defects treated with S- or L-β-TCPs showed enhanced SA-β-gal staining after one week for S-β-TCP, and after four weeks for S-β-TCP and L-β-TCP (Figure 2). SA-β-gal-positive cells are mainly found around both S-β-TCP and L-β-TCP granules. In the S-β-TCP treated group, the defects contained massive numbers of p21 positive cells. Likewise, in the L-β-TCP treated group, there were moderate numbers of p21 positive cells at one week (Figure 3). The result of p19 staining showed a similar propensity as that of p21 staining (Figure 4). The urokinase plasminogen activator receptor (uPAR) was recently identified as an indicator co-expressed with p16, representing the late senescence [43]. There were no uPAR-positive cells in bone defects among all groups for up to four weeks (Figure 5). Results indicated that the early senescent-like cells were induced after the implantation of β-TCPs in bone defects but were possibly cleared during the four weeks. 

### 2.3. Administration of Senolytics after β-TCP Implantation

**Figure 6:**
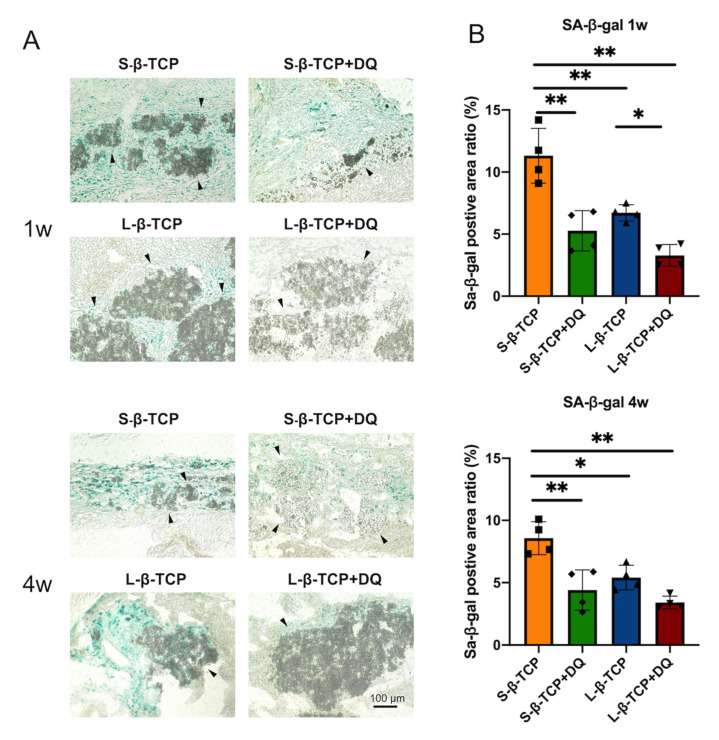
Identification of cellular senescence in bone defects at one week and four weeks after the operation. (**A**) SA-β-gal staining images of bone defects with or without DQ administration (scale bar = 100 μm). Black triangles represent β-TCP granules; (**B**) SA-β-gal positive area ratio in the bone defect. Mean with SD (*n* = 4). * *p* < 0.05, ** *p* < 0.01: One-way ANOVA analysis with Tukey–Kramer method as post hoc test. D: Dasatinib; Q: Quercetin.

**Figure 7:**
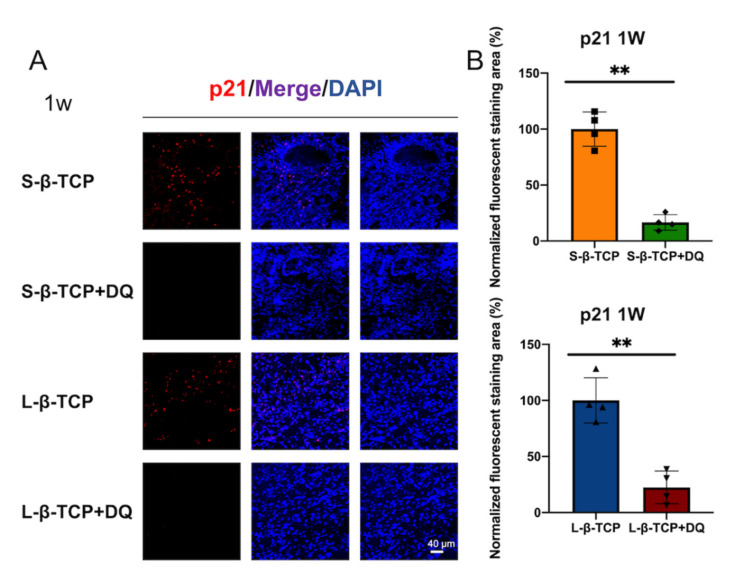
Identification of cellular senescence in bone defects one week after the operation. (**A**) Immunofluorescent images stained with p21 antibody and DAPI (scale bar = 40 μm); (**B**) p21 quantified protein level at ROI in bone defect normalized against samples without DQ. The ratio of positive area for groups without DQ administration was one hundred percent. Mean with SD (*n* = 4). ** *p* < 0.01: Student’s *t*-test. D: Dasatinib; Q: Quercetin.

**Figure 8:**
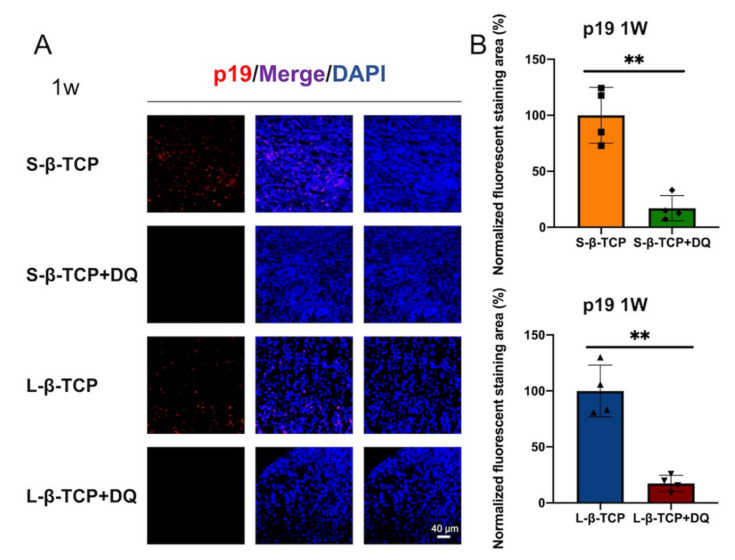
Identification of cellular senescence in bone defects one week after the operation. (**A**) Immunofluorescent images stained with p19 antibody and DAPI (scale bar = 40 μm); (**B**) p19 quantified protein level at ROI in bone defect normalized against samples without DQ. The ratio of positive area for groups without DQ administration was one hundred percent. Mean with SD (*n* = 4). ** *p* < 0.01: Student’s *t*-test. D: Dasatinib; Q: Quercetin.

Based on the results of SA-β-gal, p21, or p19 staining, we administrated senolytics (a cocktail of DQ) to decrease senescent-like cells. The level of SA-β-gal staining and of p21- or p19-positive cells in defects treated with β-TCP in parallel to oral administration of DQ was lower than those without DQ (Figure 6, Figure 7 and Figure 8).

### 2.4. Histomorphometric Analysis of the Bone Formation and β-TCP Resorption

**Figure 9:**
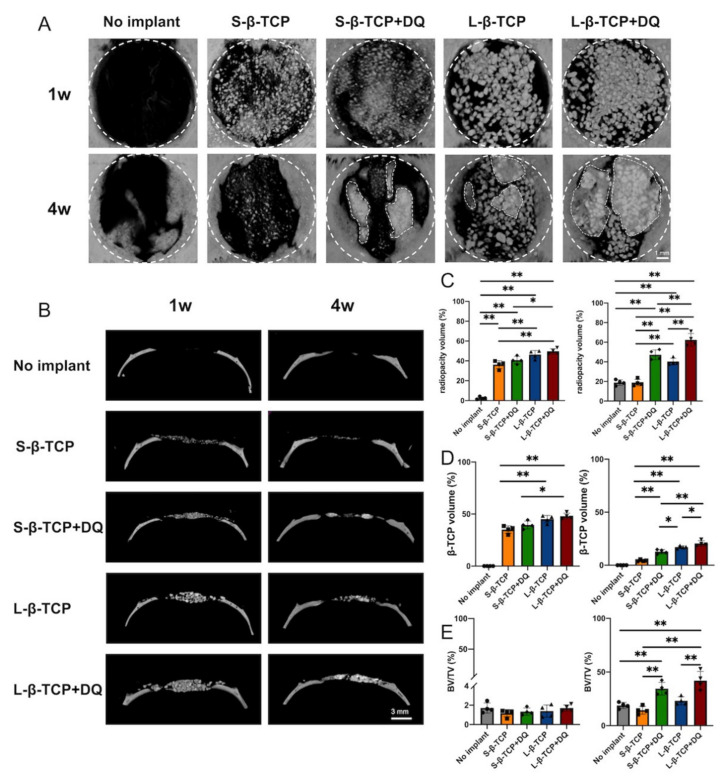
Morphometric analysis of new bone formation and residual beta-tricalcium phosphate (β-TCP) using microcomputed tomography (µCT). (**A**) Vertical microcomputed tomography. The inner part of bold broken lines represents bone defect. The inner part of thin broken lines represents the granules fused with the newly formed bone. (**B**) Lateral microcomputed tomography; (**C**) radiopacity volume in defects using µCT analysis; (**D**) volume of β-TCP residual using µCT analysis; (**E**) bone morphometry of newly formed bone by removing of β-TCP. BV: Bone volume; TV: Total volume. Mean with SD (*n* = 4). * *p* < 0.05, ** *p* < 0.01, one-way ANOVA analysis with Tukey–Kramer method as post hoc test.

**Figure 10:**
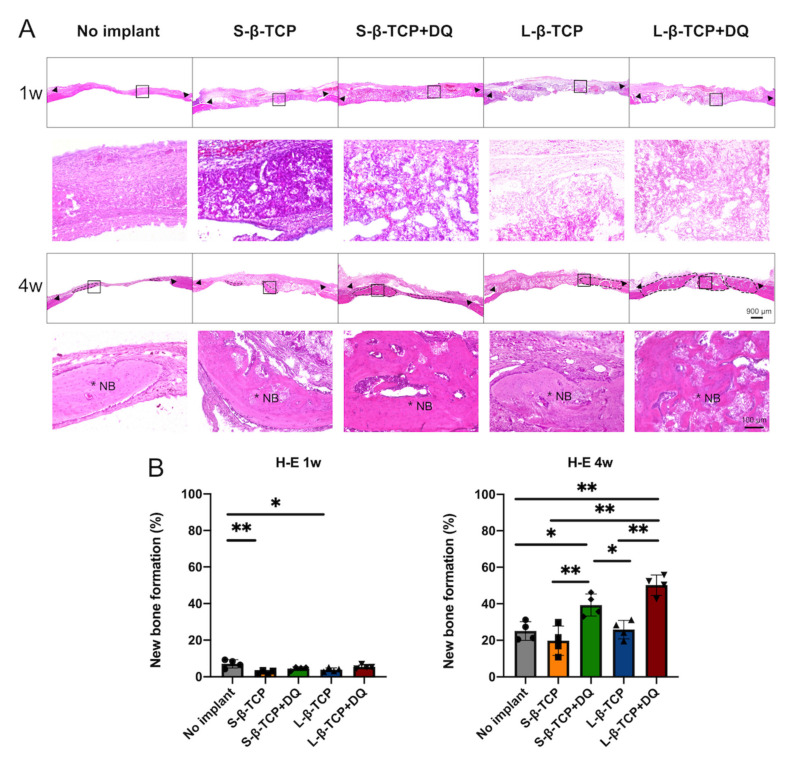
Histological evaluation of bone defects. (**A**) Hematoxylin-eosin (H-E) staining images of bone defects at one and four weeks after the operation. Upper parts represent low-magnification images (scale bar = 900 μm); lower parts represent high-magnification images of the square in low-magnification images (scale bar = 100 μm). Black broken lines represent area of newly formed bone. Black triangles represent the edge of created bone defects. * NB: New bone; (**B**) the histomorphometric analysis of newly formed bones utilizing samples of H-E staining. Mean with SD (*n* = 4). * *p* < 0.05, ** *p* < 0.01: One-way ANOVA analysis with Tukey–Kramer method as post hoc test.

Bone formation in defects was assessed using microcomputed tomography (µCT) and hematoxylin-eosin (H-E) staining (Figure 9 and Figure 10). μCT images demonstrated that the bone defects treated with L-β-TCPs showed greater radiopacity than those of S-β-TCPs up to 4 weeks. DQ administration increased radiopacity in defects compared with those treated with S- or L-β-TCP alone (Figure 9A–C). Further, the H-E staining of these bone defects showed that the increased radiopacity was due to newly formed bone (Figure 10A,B). The calculation regarding dissection of radiopacity volume of β-TCPs and histomorphometric analysis using H-E staining revealed that the bone defect treated using β-TCP with DQ oral administration expressed an increased formation of new bone in four weeks than defects treated with S-β-TCP or L-β-TCP alone (Figure 9E and Figure 10B).

In contrast, DQ administration elevated residues of β-TCP in four weeks. The amount of β-TCP granule residues in bone defects with DQ oral administration was slightly higher than those without DQ (Figure 9D).

### 2.5. Osteoclastogenesis in Bone Defects

**Figure 11:**
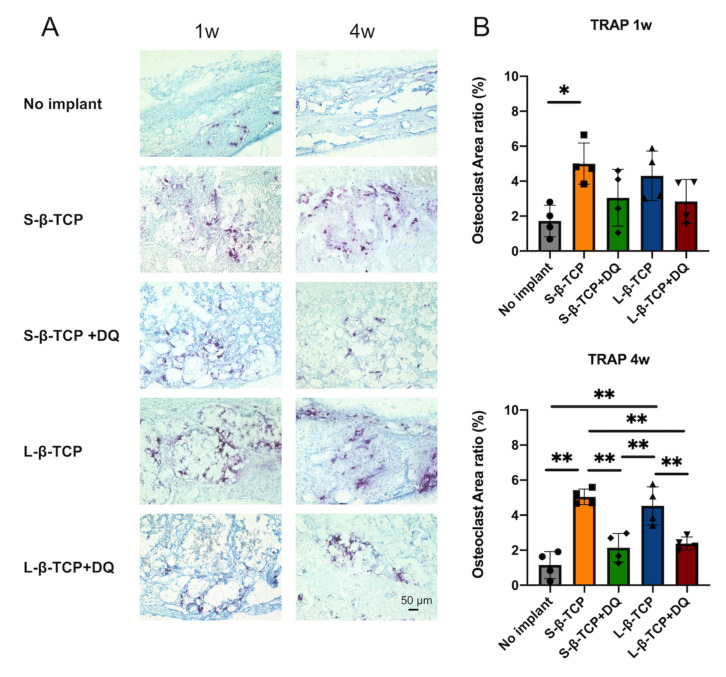
Detection of osteoclasts in bone defects. (**A**) Tartrate-resistant acid phosphatase (TRAP) staining images of the bone defect (scale bar = 50 μm); (**B**) the histomorphometric analysis of osteoclast using samples of TRAP staining. Mean with SD (*n* = 4). * *p* < 0.05, ** *p* < 0.01: One-way ANOVA analysis with Tukey–Kramer method as post hoc test.

Tartrate resistant acid phosphatase (TRAP) is a characteristic enzyme of osteoclasts associated with the resorption of bone and β-TCP [9,44]. The administration of DQ apparently led to a decrease in the number of osteoclasts in bone defects up to four weeks (Figure 11).

### 2.6. Reactive Oxidative Stress in Bone Defects

**Figure 12:**
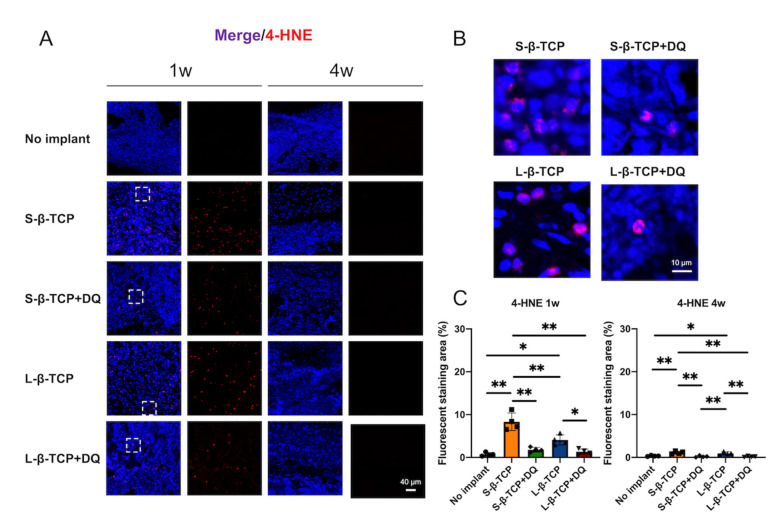
Evaluation of oxidation in bone defects. (**A**) Immunofluorescent images stained with 4-hydroxy-2-nonenal (4-HNE) antibody and DAPI after one and four weeks of operation (scale bar = 40 μm). Broken squares represent areas magnified in (**B**); (**B**) magnified view of the areas in (**A**) (scale bar = 10 μm); (**C**) 4-HNE quantified level at ROI in the bone defect. 4-HNE-positive area against nucleus area. Mean with SD (*n* = 4). * *p* < 0.05, ** *p* < 0.01: One-way ANOVA analysis with Tukey–Kramer method as post hoc test.

A major biomarker giving rise to reactive oxidative stress (ROS) is the presence of 4-hydroxy-2-nonenal (4-HNE) [45]. Bone defects treated with S-β-TCP and L-β-TCP showed a significant rise in their 4-HNE positive cells, whereas the ones treated using S-β-TCP and L-β-TCP with DQ administration showed fewer 4-HNE positive cells after a week (Figure 12).

## 3. Discussion

The present study demonstrated that the implantation of β-TCP granules induced senescent cells in critical-sized bone defects in rat calvaria; the oral administration of DQ to rats, implanted with β-TCP granules, markedly inhibited cellular senescence induction mechanisms and promoted bone regeneration.

In general, SA-β-gal staining is a biomarker to detect cellular senescence [46]. However, its reliability when applied in the field of bone biology is controversial. In addition to senescent cells, macrophages [47] or osteoclasts [41,48] with hyper-functional lysosomes are stained positively by SA-β-gal [49,50]. Material consisting of small granules elicits an inflammatory response in surrounding tissues via macrophages [51]. β-TCP is absorbed by osteoclasts when implanted in bone defects [52]. The SA-β-gal staining was stronger in S-β-TCP granules than in L-β-TCP granules at one week and four weeks. In addition, administration of DQ failed to decrease all the numbers of cells stained with SA-β-gal staining (Figure 6). These results suggest that SA-β-gal staining in this study is likely to stain cells such as macrophages and osteoclasts as well as senescent cells.

There have been only a few reports of material implants responsible for the induction of senescent cells, except for those by silicone breast implants [53]. In this study, β-TCP granules around the implanted β-TCPs showed a significant enhancement in the staining of p19, p21, and SA-β-gal after one week, whereas no p19- or p21-positive cells were found at four weeks. Several cells stained with uPAR (an indicator co-expressed with p16) were identified at either one week or four weeks. De Cecco et al., reported that p21 and p16 indicate early cellular senescence and late cellular senescence, respectively [54]. These results imply that the implantation of β-TCP may induce early cellular senescence, yet senescent cells might fail to reach the late senescence stage.

β-TCP granules are reported to induce the production of ROS [13,14]. The water-soluble granules could lead chemical stress to the surrounding tissues during dissolution [13,55] and mediate the production of osteoclasts [56]. These reasons can elicit significant ROS production. In this study, the staining of 4-HNE, an indicator of ROS, was stronger at one week. ROS coincides with the results of p21 and p19 staining. Increased levels of ROS in tissues lead to a loss of adaptive response and towards the progression of cellular senescence [57,58]. p21 levels in senescent cells are usually induced by ROS, which reflects DNA damage [59]. Although the complete mechanism of β-TCP granules inducing senescent cells is still unknown, the ROS generated by β-TCP granules may be partly responsible for the appearance of p19- and p21-positive senescent cells.

Considering that two sizes of β-TCP granules used in this study induced senescent-like cells, senolytics (a cocktail of DQ) were used to evaluate the effect of senescent cells on local bone regeneration with implanted β-TCP. Oral administration of DQ significantly decreased p19 and p21 staining, reduced the levels of ROS, and increased bone formation in bone defects treated with both S-β-TCP and L-β-TCP. To the best of the authors’ knowledge, these results are the first evidence that DQ stimulates bone formation with implantation of β-TCP, possibly by the mechanism of inhibiting cellular senescence around granules.

**Figure 13:**
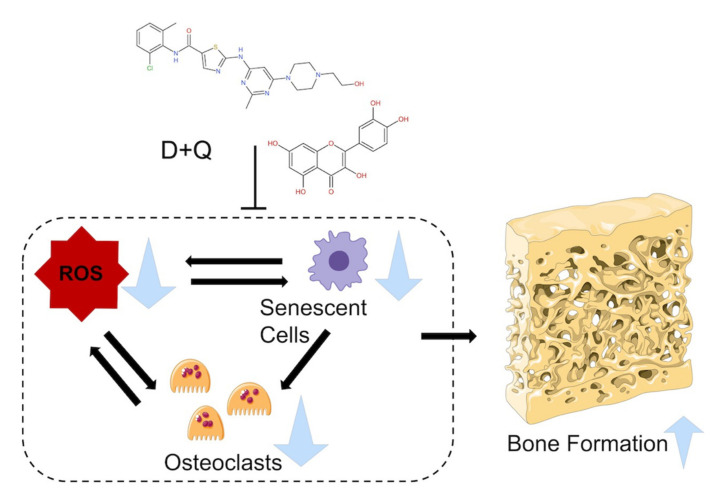
Hypothetical scheme for boosting bone formation ability of beta-tricalcium phosphate (β-TCP) by senolytic administration.

This study infers that DQ administration retarded the resorption of both β-TCPs, possibly by the inhibition of osteoclastogenesis (Figure 9D and Figure 11). Bone formation apparently increased after DQ administration to the defects treated with S- and L-β-TCPs. (Figure 9 and Figure 10). There might be some possibility that DQ participated in bone regeneration through the direct activation of osteoblasts. However, dasatinib has been reported to inhibit the proliferation of mesenchymal stem cells and osteoblastic differentiation in vitro [60]. Meanwhile, previous studies by other groups have reported that senescent cells can promote the formation of osteoclasts and ROS [59,61,62,63]. Increased levels of ROS promote the formation of senescent cells and osteoclasts [64,65]. β-TCP is known to initiate bone reconstruction by chemical degradation and resorption by osteoclasts [66,67], while large numbers of osteoclasts and rapid resorption of β-TCP are known to inhibit bone formation to a certain extent [68,69]. The optimal scaffold provides mechanical support for bone regeneration and facilitates cell attachment, proliferation, and differentiation [70,71]. Given these results, DQ may have assisted in the gradual resorption of β-TCP granules through the reduction of senescent cells and ROS levels that lead to the inhibition of osteoclast formation and partially provide an adequate scaffold for bone formation (Figure 13).

In this study, the implantation of β-TCP granules surged the number of senescent cells; implanted β-TCP with the oral administration of DQ resulted in more significant bone formation than the implantation of β-TCP alone. However, the complete mechanisms underlying the β-TCP granules to cause cellular senescence, such as the specific location of the cells and spatial-temporal relation between ROS and senescent cells, remain unclear. In addition, complete mechanisms to enhance bone formation by DQ administration remain unsolved. However, the results of this study may provide insight to enhance bone formation using β-TCP, which is extensively used in dental and orthopedic applications. These data would aid in establishing new CaP-based bone regeneration therapies (such as periodontal tissue regenerative therapy and maxillofacial surgery, etc.) and in further development of CaP-based materials.

## 4. Materials and Methods

### 4.1. β-TCP Granules

β-TCP granules (Taihei chemical industrial Co., Osaka, Japan) were divided into S-β-TCP (below 300 μm) and L-β-TCP (300–500 μm) based on their diameter. S-β-TCP and L-β-TCP granules were screened out using 300 μm and 500 μm sieves (Cat. No.: 5-3291-33, 5-3291-36, As one Co., Osaka, Japan). All granules were polished under mortar and pestle.

### 4.2. Characterization of β-TCP Granules

The camera used to capture material observation images was the Canon EOS 600D (Canon Inc., Tokyo, Japan). After the surface of β-TCP granules was coated with osmium by HPC-20 Osmium Coater (Vacuum Device Inc., Ibaraki, Japan), FE-SEM (S-4800, Hitachi Co., Tokyo, Japan) was used to confirm the β-TCP surface structure, porous structure, and particle size. Images from FE-SEM were taken at 5.0 kV and 10.0 μA. The elemental analysis of the samples was determined by XPS (PHI X-tool; ΦULVAC-PHI, Inc., Kanagawa, Japan). XRD (XRD-6100, Shimadzu Co., Kyoto, Japan) was used to determine the crystal structure of the β-TCP at 30.0 mA, 40.0 kV Cu-Ku radiation. The scanning rate was 2°/min and the 2θ range was 10–60°. Using ATR-FTIR (IRAffinity-1S, Shimadzu Co., Kyoto, Japan), the S-β-TCP and L-β-TCP granules were scanned to confirm chemical bonds. The number of scans was 16. Adjustment of the baseline and smoothing were used.

### 4.3. The Preparation of the Senolytics Using Dasatinib and Quercetin

Polyethylene Glycol 200 (PEG-200, Cat. No.: ESL3444, FUJIFILM Wako Pure Chemical Co., Osaka, Japan) was dissolved in MillQ to make the PEG-200 solution. Dasatinib (Cat. No.: 11498, Cayman Chemical Co., Ann Arbor, MI, USA) and quercetin (Cat. No.: sc-206089A, SCB Santa Cruz Biotechnology Inc., Dallas, TX, USA) were dissolved in a PEG-200 solution and mixed well with Voltex to make senolytics. The senolytics were orally administrated at 6.67 mg/kg (D) and 66.7 mg/kg (Q) for each rat.

### 4.4. Animal Experiment

Since senescent cells accumulate with age, young Sprague Dawley rats (male, 8 weeks old) were used as the experimental group for the purpose of avoiding the influence of the excess creation of age-related senescent cells. Rats were purchased from SHIMIZU Laboratory Supplies Co. (Kyoto, Japan). Pre-operative anesthesia was performed by injecting three types of mixed anesthetic agents (Butorphanol tartrate, Midazolam, and Domitor) into their peritoneal cavity. A critical-sized defect (9 mm in diameter) was made in the center of the calvaria of rats individually utilizing a trephine bar (Dentech Co., Tokyo, Japan). The defect was filled uniformly with 30 mg of β-TCP granules. The periosteum and skin were covered and tightly sutured to ensure the granules were stable. Rats were divided into the following groups: 1, no implant as the negative control group; 2, implanted with β-TCP granules below 300 μm; 3, implanted with β-TCP granules below 300 μm and fed with DQ senolytics; 4, implanted with β-TCP granules 300–500 μm; and 5, implanted with β-TCP granules 300–500 μm and fed with DQ senolytics (four rats per group). Experimental groups 3 and 5 were administered senolytics on the day of surgery with a sonde, followed by once a week for four weeks. One or four weeks after implantation, rats were euthanized, and their calvarias were collected as samples. Samples were stored in a 4% paraformaldehyde phosphate buffer solution (Cat. No.: LEM5621, FUJIFILM Wako Pure Chemical Co., Osaka, Japan) at 4 °C. All animal experiments were permitted by and strictly complied with the policy of the Local Ethics Committee of Osaka Dental University (Approval No.: 21-02015).

### 4.5. The Analysis of Bone Histomorphometry Utilizing Microcomputed Tomography

Fixed samples were scanned utilizing microcomputed tomography (µCT) (SkyScan 1275, Bruker Co., Billerica, MA, USA) at 85 kV and 70 µA. SkyScan™ CT analyzer software (version 1.17.7.2) was then used to synthesize images and measure quantitative data of morphological parameters to observe bone formation and morphology. The following parameters were quantified: Radiopacity volume ((radiopacity volume/total volume of defect) × 100), BV/TV ((bone volume/total volume of defect) × 100).

### 4.6. Histological Staining

Samples were divided into halves. The left halves of samples were decalcified utilizing decalcifying solution B (Cat. No.: LEP2494; FUJIFILM Wako Pure Chemical Co., Osaka, Japan). Counterparts were kept undecalcified. All the decalcified and undecalcified samples were embedded using the Kawamoto method and 4-µm-thick frozen sections were obtained using cryotome (Leica CM3050S; Leica Biosystems Co., Richmond, IL, USA) [72]. Decalcified sections underwent H-E staining to visualize osteogenesis, TRAP staining to visualize osteoclasts, and undecalcified sections underwent SA-β-gal staining (Cat. No.: ab65351, abcam, Cambridge, MA, USA) to visualize senescent cells. Images were taken by a BZ-9000 digital microscope (Keyence Corp., Osaka, Japan). H-E staining images at four weeks were taken in the bone-forming area. Histomorphometric analysis was carried out using Adobe Photoshop Elements (Adobe Systems Inc., San Jose, CA, USA) and ImageJ (version: 2.1.0, U.S. National Institutes of Health, Bethesda, MD, USA). The SA-β-gal-positive cells ratio in the defect was calculated in accordance with the following formula: (positive cells area/complete tissue area in defect) × 100. The proportion of the newly formed bone in the defect was calculated in accordance with the following formula: (newly formed bone area/complete tissue area in defect) × 100. The proportion of osteoclast area in the defect was calculated in accordance with the following formula: (TRAP-positive area/complete tissue area in defect) × 100 [73,74].

### 4.7. Immunofluorescence

Immunofluorescent staining was performed to observe cellular senescence and ROS. The decalcified sections obtained in Section 4.6 were blocked and permeabilized with 5% goat serum, 0.3% Triton X-100 in PBS. Next, sections were incubated with a primary antibody (Anti-CDKN2A/p19ARF Polyclonal Antibody, ALEXA FLUOR^®^ 555 Conjugated (Cat. No.: bs-1174R-A555, Bioss Antibodies Inc, Woburn, MA, USA), Anti-P21 Polyclonal Antibody, ALEXA FLUOR^®^ 555 Conjugated (Cat. No.: bs-10129R-A555, Bioss Antibodies Inc., Woburn, MA, USA), or Anti-4 Hydroxynonenal Polyclonal Antibody, ALEXA FLUOR^®^ 647 Conjugated (Cat. No.: bs-6313R-A647, Bioss Antibodies Inc., Woburn, MA, USA)) overnight at 4 °C and then mounted with DAPI-Fluoromount-G^®^ (Cat. No.: 0100-20, Southern Biotechnology Associates Inc., Birmingham, AL, USA). Sections were observed under a laser confocal microscope (LSM-700, Zeiss Microscopy, Jena, Germany). Immunofluorescent staining sections were horizontally divided into the left, center, and right and vertically divided into top and bottom. As the left and right sides were symmetrical, the extracted region of interests (ROI) were quantified under the following conditions: Upper left, lower left, upper middle, lower middle, one each. Images were analyzed with ImageJ to assess the ratio of positive staining area of p19, p21, and 4-HNE to the area of DAPI ((antibodies positive staining area/DAPI area) × 100), and then the average ratio of the four images was calculated by assessing a single sample. The sections obtained in Section 4.6 were blocked with 1% BSA. Sections were incubated with a primary antibody (uPAR antibody (Cat. No.: DF-12495, Affinity Biosciences, Jiangsu, China)) overnight. Then, sections were incubated with a secondary antibody (Goat Anti-Rabbit IgG H&L Alexa Fluor^®^ 594 (Cat. No.: ab150080, abcam co., Cambridge, MA, USA)) for 1 h and mounted with DAPI-Fluoromount-G^®^.

### 4.8. Statistical Analysis

All results were declared as the mean ± standard deviation (SD). For contrasting between the four groups, a one-way analysis of variance (ANOVA) was carried out. For contrasting between two groups, student’s *t*-test was executed. If the ANOVA results were significant, the Tukey–Kramer method was used as a post hoc test. All statistical analyses were carried out using Prism 8 (GraphPad Software Co., San Diego, CA, USA).

## 5. Conclusions

The present study demonstrated that the implantation of β-TCP induces the formation of senescent-like cells—SA-β-gal, p21-, and p19-potitive cells—in critical-sized bone defects in rat calvaria. However, the uPAR-positive senescent cells co-expressed with p16 could not be found up to 4 weeks. The implantation of β-TCP also increased ROS production and osteoclast growth in defects. The oral administration of DQ reduced those senescent cells, the levels of ROS, and the number of osteoclasts. In addition, defects with β-TCP implantation and DQ administration showed more significant bone formation than in those without DQ. These results suggest that cellular senescence mechanisms might attenuate the bone-forming ability of β-TCP. Although a further detailed examination is inevitable, modulation of the cellular senescence mechanisms is likely to boost the bone-forming ability of β-TCP. These data will provide fundamental insights in bridging the cellular senescence and bone regeneration and contribute to the development of novel biomaterials in regenerative medicine.

## Data Availability

Not applicable.
